# Multimodality assessment of aortic stenosis severity in Transcatheter Aortic Valve Implantation (TAVI): comparison between cardiovascular magnetic resonance, transesophageal and transthoracic echocardiography

**DOI:** 10.1186/1532-429X-14-S1-O70

**Published:** 2012-02-01

**Authors:** Andrew Jabbour, Tevfik F Ismail, Ali Vazir, Isabelle Roussin, Ankur Gulati, Francisco Alpendurada, Sameer Zaman, Oluwatosin Sotubo, Saman S Zaman, Benjamin Hewins, Simon Davies, Sanjay K Prasad, Susanna Price, Michael B Rubens, Neil Moat, Raad H Mohiaddin

**Affiliations:** 1 NIHR Cardiovascular Biomedical Research Unit, Royal Brompton and Harefield NHS Foundation Trust, Imperial College London, London, UK; 2 Cardiothoracic Surgery, Royal Brompton and Harefield NHS Foundation Trust, London, UK; 3 Intensive Care Unit, Royal Brompton and Harefield NHS Foundation Trust, London, UK; 4 Radiology, Royal Brompton and Harefield NHS Foundation Trust, London, UK

## Background

Although transthoracic echocardiography (TTE) is the initial and best validated modality for the assessment of aortic valve stenosis severity, both cardiovascular magnetic resonance (CMR) and transesophageal echocardiography (TEE) provide valuable complimentary information, particularly when patients are being assessed for Transcatheter Aortic Valve Implantation (TAVI). It is increasingly recognised that aortic valve area normalised to body surface area may better represent the severity of aortic stenosis than estimates derived from measurements of peak flow velocity as these can be load-dependent. In addition, Doppler-derived measurements may be limited by errors arising from misalignment of Doppler jet and poor windows. This study sought to determine the agreement and variability between CMR-, TEE- and TTE-derived measures of aortic stenosis severity.

## Methods

One hundred and twenty three patients assessed by CMR and TTE prior to TAVI were studied. Aortic-valve stenosis severity was assessed by TTE with traditional Doppler measurements and computed using the continuity equation. By CMR, the severity of aortic stenosis was assessed using a contiguous stack of steady-state free precession (SSFP)-based, small field of view, short axis cines (6mm slice thickness, no gap) and quantified by planimetry of aortic valve area (AVA) at peak-systole (CMRtools, Cardiovascular Imaging Solutions, London UK). TEE, performed just prior to TAVI insertion, was also used to measure AVA by planimetry. Agreement and variability between each imaging modality were assessed by Bland-Altman analysis.

## Results

Of one hundred and twenty three patients assessed by CMR and TTE prior to TAVI, 55 also underwent TEE. Close agreement and almost negligible bias was observed between CMR- -derived planimetry of AVA and TTE-continuity-equation-derived AVA (0.06 (0.27) cm^2^; -0.47 cm^2^ to 0.58 cm^2^, (Bias (SD of Bias); 95% Limits of agreement)). CMR- and TEE-based planimetry also showed close agreement with no clinically significant bias (0.096 (0.33) cm^2^; -0.54 cm^2^ to 0.73 cm^2^).

## Conclusions

Planimetry of aortic valve area in patients with severe aortic stenosis by CMR shows close agreement and negligible bias when compared with continuity equation-derived AVA measures by transthoracic echocardiography. Furthermore, CMR and transesophageal echocardiography-derived AVA by planimetry also show close agreement. AV planimetry is important in the assessment of aortic stenosis severity in TAVI patients undergoing CMR.

## Funding

This project was supported by the NIHR Cardiovascular Biomedical Research Unit of Royal Brompton and Harefield NHS Foundation Trust, the British Heart Foundation, and CORDA. Dr. Jabbour was supported by a Postdoctoral Research Fellowship from the National Health and Medical Research Council of Australia, a Vincent Fairfax Family Foundation Research Fellowship from the Royal Australasian College of Physicians, the St. Vincent’s Clinic Foundation, and the Victor Chang Cardiac Research Institute.

